# Monkeypox Virus Infections in Small Animal Models for Evaluation of Anti-Poxvirus Agents

**DOI:** 10.3390/v2122763

**Published:** 2010-12-20

**Authors:** Christina L. Hutson, Inger K. Damon

**Affiliations:** Poxvirus and Rabies Branch, Centers for Disease Control and Prevention, 1600 Clifton Rd. MS-G06 Atlanta, GA 30029, USA; E-Mail: zuu6@cdc.gov

**Keywords:** monkeypox, animal models, therapeutics

## Abstract

An ideal animal model for the study of a human disease is one which utilizes a route of infection that mimics the natural transmission of the pathogen; the ability to obtain disease with an infectious dose equivalent to that causing disease in humans; as well having a disease course, morbidity and mortality similar to that seen with human disease. Additionally, the animal model should have a mode(s) of transmission that mimics human cases. The development of small animal models for the study of monkeypox virus (MPXV) has been quite extensive for the relatively short period of time this pathogen has been known, although only a few of these models have been used to study anti-poxvirus agents. We will review those MPXV small animal models that have been developed thus far for the study of therapeutic agents.

## 1. Introduction

An ideal animal model for the study of a human disease is one which utilizes a route of infection that mimics the natural transmission of the pathogen; the ability to obtain disease with an infectious dose equivalent to that causing disease in humans; as well having a disease course, morbidity and mortality similar to that seen with human disease. Additionally, the animal model should have a mode(s) of transmission that mimics human cases. Factors which subsequently allow more detailed inferences about disease pathogenesis include the availability of reagents to evaluate host innate and adaptive immune responses to the pathogen, and histopathological changes in the host which result from infection or the host response to infection. These findings can then be compared to what is known of human disease. The utility of a small animal model of human disease for study of therapeutic efficacy is augmented when large numbers of animals are available for use in appropriately, well-powered studies. Even if all aspects of an animal model of disease are not completely faithful to what is known of human disease, important information regarding therapeutic efficacy can be gleaned from their use in “pre-clinical” studies.

The published literature on clinical manifestations of systemic human orthopoxvirus disease is derived from historic literature descriptions of human smallpox and more recent descriptions of human monkeypox disease. The clinical-descriptive literature on human monkeypox is expected to grow in the next five years, as data acquisition and analysis from an ongoing study in the Democratic Republic of Congo is finalized. Currently available literature is largely derived from WHO-sponsored surveillance efforts in West Africa and the Congo Basin in the 1980s, after the first recognition of human disease in these areas, and subsequent analyses of public health response data and human research studies following the introduction of West African clade virus into the U.S. in 2003. Human monkeypox, as described through the active surveillance and case ascertainment studies sponsored by WHO in the 1980s, was depicted as resembling discrete ordinary smallpox. In natural human infection, exposure leading to infection is believed to occur via a respiratory route, with subsequent progressive viremias/lymphemia, ultimately leading to seeding of the skin to generate a generalized rash. Percutaneous exposure, also leading to generalized rash formation, has also been described for both viral infections. The disease pathogenesis has been conjectured and modeled largely from animal studies; initial models were using ectromelia infection of mice; some kinetic observations of virus shedding and viremia have been made in human studies of smallpox and monkeypox. The time course of disease is generally thought to include an asymptomatic phase of 10–12 days, during which time the virus initially enters the host, replicates, seeds reticuloendothelial organs, replicates, then spreads via the bloodstream (inducing a febrile response) which is the first symptomatic hallmark of disease. The fever is usually described as occurring 10–12 days post initial exposure/infection. The range has been 7–17 days. Fever is accompanied by other symptoms, including headache, backache, myalgias, and or abdominal pain. Two to three days following the fever, rash develops—initially presenting as a macular, then papular, then vesicular and pustular eruption. Scabbing then begins. Each stage of rash lasts 1–2 days. Approximately 2–3 weeks post initial symptoms, scabs begin to separate from the skin. Death and disease severity have had some correlation with rash burden in epidemiologic studies of hospitalized smallpox patients. Severe outcomes are more frequent in unvaccinated, younger age groups; death occurs within the first week of illness in cases with hemorrhagic manifestations, and during the second or third week of illness in “ordinary” cases. In the human monkeypox cases studied in Zaire/DRC between 1981–1986, of the 33 deaths among 338 patients, all occurred in unvaccinated children less than eight years of age. Death occurred during the first week of illness in 21%, the second week in 52%, and the third week of illness in the remaining 27% [1].

The development of small animal models for the study of monkeypox virus (MPXV) has been quite extensive for the relatively short period of time this pathogen has been known. Initial animal models were designed to address natural history in potential or surrogate reservoir host species, as well as studies of disease in primates. Routes of exposure were designed to evaluate disease if respiratory or percutaneous exposures occurred, or in some cases to simply address whether virus would replicate in the animal model system. Factors that influence the outcome of a challenge study include the age of the animal at time of infection, inoculation route used, and the viral dosage given. Additionally, the strain of MPXV (currently delineated as belonging to Congo Basin clade or West African clade) used in the study may influence the disease severity.

## 2. Animal Models Using Monkeypox Virus as a Challenge Virus (Table 1)

Guinea pigs and golden hamsters were found to be relatively resistant to MPXV (West African clade Copenhagen strain) infection by multiple routes. Guinea pigs were challenged via an intracardial, intranasal (IN), oral or foot pad (FP) inoculation with no observable symptoms of disease except for edema at the FP inoculation site. Golden hamsters were also resistant to MPXV infection via several routes of infection with no observable signs of disease, even with large dosages of virus (1.5–5.9 × 10^7^) [2].

Rabbits have also been considered as a possible animal model for the study of MPXV [2–3]; susceptibility depended greatly on the method of inoculation and the age of the animals. In adult rabbits challenged with MPXV (West African clade Copenhagen strain) via an oral inoculation, no signs of disease were seen. However, if virus was delivered by intravenous route, acute illness was observed with generalized rash. Young rabbits (10 days old) inoculated via IN or oral route developed severe illness; two day old rabbits were highly susceptible to infection by intracutaneous inoculation or skin scarification [4]. The intracutaneous route led to the development of discrete white translucent lesions. Another study found that intracerebral inoculation was 100% fatal and that intratesticular or intracorneal inoculation with MPXV was also pathogenic in rabbits [5].

Several rat species including white rats, cotton rats and multimammate rats have been challenged with MPXV. Adult white rats inoculated with 10^1^ to 10^3^ plaque forming units (pfu) of West African MPXV were not susceptible to infection with intravenous, IN, or cutaneous routes of infection. However, newborn white rats (1–3 days old) developed adynamia leading to death in 5–8 days when challenged with MPXV intranasally [2]. Cotton rats and multimammate rats were both found to be highly susceptible to MPXV infection. When cotton rats were challenged with 10^5^ pfu via an intravenous route of infection, 100% mortality was seen 4–5 days post infection (p.i.). The infection was characterized by difficulty breathing, cough, sneezing, cyanosis, rhinitis, purulent conjunctivitis, and progressive emaciation. An IN MPXV challenge in cotton rats caused 50% mortality with a clinical picture such as that seen with the intravenous route [6,7]. Multimammate rats were highly sensitive to both IN and intraperitoneal (IP) inoculation [7,8].

Marennikova *et al.* challenged adult common squirrels (*Sciurus vulgaris*) with 10^6^ pfu of MPXV Z-249 (Congo Basin clade) via IN, oral or scarification routes of infection [9]. Disease progression occurred earlier in animals infected IN or orally than those animals infected via a scarification route. Skin lesions did not develop on any animals; symptoms of disease included fever, inactivity, inappetence, rhinitis, cough and difficulty breathing. Infection was 100% lethal by day 7 or 8 p.i. regardless of inoculation route. Shelukhina *et al.* challenged six African squirrel species (including members of the genera *Funisciurus*, *Protexerus* and *Heliosciurus*) with Congo Basin MPXV via an IN infection (10^5^ or 10^6^ pfu/0.1 mL) [10]. All squirrel species were highly susceptible to Congo Basin MPXV challenge and developed an acute, generalized infection that was 100% lethal. However, some varying degree of susceptibility in the different squirrel species was seen with lesser dosages of virus. Cutaneous inoculation of squirrels resulted in a thick, red papule at the inoculation site. Skin lesions (restricted to non-fur-bearing areas of the skin or at the borders of the skin and mucous membranes of the nose and lip) occurred in only a few squirrels that had been infected by the oral or IN route with small (nonlethal) doses of virus. Most often the rash appeared in the later stages of disease (16–25 days p.i.). Transmission studies were also conducted with squirrels and authors found that infected animals were able to transmit the disease to naïve animals via airborne and direct contact [10].

Ground squirrels (*Spermophilus tridecemlineatus*) are very susceptible to MPXV infection. Tesh *et al.* challenged adult ground squirrels with the West African MPXV clade either IP or IN with 10^5.1^ pfu [11]. In both groups, symptoms of disease included anorexia and lethargy within 4–5 days of infection, with no other observable symptoms. Weight loss was not measured for these animals. Animals in the IP group died within 6–7 days p.i.; those IN challenged all died within 8–9 days. A follow-up study compared the pathogenesis of the two MPVX clades in the ground squirrel model [12]. Inoculation of 100 pfu by a subcutaneous route of infection was 100% lethal for both MPXV clades. However, the authors noted that the onset of severe respiratory distress was more rapid and uniform for the Congo Basin MPXV challenged animals. Additionally, animals challenged with the Congo Basin MPXV began to die earlier than West African challenged animals. However, LD_50_ values were similar for the two strains using the ground squirrel MPXV model (0.35 pfu for Congo Basin and 0.46 pfu for West African MPXV).

Prairie dogs have also been looked at as a possible MPXV animal model. Xiao *et al.* challenged prairie dogs with 10^5.1^ pfu of West African MPXV via an IP or IN route of infection and observed 100% and 60% mortality, respectively [13]. Hutson *et al.* challenged prairie dogs with either the Congo Basin or West African MPXV (10^4.5^ pfu) via an IN or scarification route of infection [14]. Animals were asymptomatic until days 9–12 when a generalized rash was observed on challenged animals. Signs of disease included lethargy, inappetence, nasal discharge, respiratory distress, and diarrhea; morbidity was noticeably more for the Congo Basin MPXV challenged animals as was mortality. A follow-up study found the LD_50_ for the prairie dog MPXV model is approximately a hundred-times lower for the Congo Basin clade compared to the West African clade (5.9 × 10^3^ and 1.29 × 10^5^, respectively) [15], utilizing an IN route of infection. Weight loss occurred in 2/4 West African MPXV challenged dosage groups and 3/4 Congo Basin MPXV challenged dosage groups; for both viral strains the highest percent weight loss calculated occurred in the highest viral inoculum group. A trend of increasing viral titers in oropharyngeal swabs with increasing viral inoculums dose was apparent for both MPXV strains, and when all mean values were combined, Congo Basin challenged animals had statistically higher levels of virus. Furthermore, the duration of MPXV DNA and viral shedding tended to occur earlier, attain higher levels, and persist longer for Congo Basin challenged animals. Symptoms were also more numerous and severe for Congo Basin MPXV infected prairie dogs.

Schultz *et al.* challenged African dormice, *Graphiuris kelleni*, with 1.4 × 10^4^ pfu of a Congo Basin clade of MPXV via FP route and observed 92% mortality [16]. The authors further developed the model by infecting dormice with various dosages of Congo Basin MPXV by the IN route and calculated the LD_50_ as 12 pfu. Animals became symptomatic at day 3 (conjunctivitis and dehydration), and animals that succumbed to disease had a mean time to death of 7.9 ± 1 to 12.3 ± 5 days (depending on dose). Morbidity for those animals that succumbed to disease included decreased activity, hunched posture, unkempt hair coat, dehydration, and conjunctivitis; lesions did not develop on any animals. Weight loss was highest with 2,000 pfu (the highest dosage given), but also was seen in animals given 200 or 20 pfu. Weight loss was not observed in the lowest dosage groups (2 and 0.2 pfu). Disease pathogenesis was described as localized inflammation, viral replication and hemorrhage in the nasal mucosa, followed by dissemination around day 3 with subsequent necrosis of liver, spleen, lung and gastrointestinal tract tissues. When the West African strain of MPXV was used to challenge dormice by an IN infection, similar days until death and mortality rates were seen as the Congo Basin MPXV challenged animals.

As is the case for many pathogens, mice have been utilized numerous times for the study of MPXV. Results have varied greatly depending on the type of mice used (*i.e.*, wild strains or laboratory strains). In early studies [2,4] white mice were challenged via intracerebral, IN, IP, FP, oral or intradermal (ID) inoculation with a West African strain of MPXV and found to be highly sensitive to most inoculation routes. Intracerebral inoculation was 100% fatal in adult white mice as was IN inoculation of suckling mice [4]. Inoculation in eight day old mice via the FP, IP, or IN route resulted in 100% mortality; ID or oral inoculation caused 50% and 40% mortality, respectively. Oral inoculation of 12 day old white mice only caused 14% mortality; however IN inoculation in 15 day old animals led to 100% mortality [2]. Inbred laboratory mouse strains have also been studied by several groups. Hutson *et al.* compared the two clades of virus (10^5^ pfu) in immunocompetent BALB/c and C57BL/6 laboratory mouse strains via an IN or FP route of infection [17]. Localized signs in the FP challenged animals included edema at the inoculation site, while the Congo Basin IN route of infection led to weight loss. However, symptoms were minimal and all animals survived infection. Osorio *et al.* compared both clades of virus by an IP inoculation in either BALB/c or severe combined immune deficient (SCID) BALB/c mice [18]. Biophotonic imaging was used to visualize the disease progression. BALB/c mice developed rough coats, and decreased activity but cleared infection within 10 days p.i. In contrast, SCID BALB/c mice developed similar symptoms, but resulted in 100% mortality by day 9 p.i. (Congo Basin MPXV) or day 11 p.i. (West African MPXV). Stabenow *et al.* utilized a laboratory mouse strain lacking STAT1 (C57BL/6stat-/-), an important protein involved in Type I and Type II IFN signaling [19]. Mice were challenged with dosages between 4.7 to 4,700 pfu via an IN route of infection. Weight loss was seen with all dosages given except for the lowest (4.7 pfu). Mortality occurred at 25–50% at the 47 pfu dose (12–21 days p.i.); 100% mortality occurred by day 9 p.i. with the highest dose given (4,700 pfu). The calculated LD50 for the Congo Basin clade in the C57BL/6stat-/- mice was 213 and 47 pfu for females and males, respectively. Americo *et al.* screened 38 inbred mouse strains and identified three that are highly susceptible to MPXV [20]. Of these three strains, the CAST/EiJ was developed as a model. Signs of morbidity in moribund animals included ruffled fur, hunched posture, and lethargy; no animals developed lesions. Animals challenged with the highest dosages by an IN route (10^5^ or 10^6^ pfu Congo Basin MPXV) lost up to 28% of the starting body weight and 100% died between days 5–8. Animals challenged with 10^4^ pfu all died between days 8–10. Animals challenged with 10^3^ pfu had an even longer delay in weight loss and death and 40% of the animals recovered. No animals perished in the 10^2^ pfu challenge group. The calculated LD_50_ for the Congo Basin clade in the CAST/EiJ mice given an IN challenge was 680 pfu. Animals were found to be even more sensitive with an IP Congo Basin MPXV infection and the calculated LD_50_ was 14 pfu. Challenging mice with 10^5^ or 10^6^ pfu of a West African MPXV strain resulted in rapid weight loss and 100% mortality by day 8 p.i. Lower dosages of West African MPXV resulted in less weight loss and lower amounts of death than what was observed for the Congo Basin MPXV; the calculated LD_50_ was 7,600 pfu, more than a log higher than for the Congo Basin clade.

## 3. Antiviral Protection in Small Animals Challenged With Monkeypox Virus

To date, four MPXV small animal models have been used for the testing of antiviral drugs Cidofovir, CMX001 and ST246 (tecovirimat). Herein we will summarize those studies, efficacy data, and discuss the advantages, and limitations, of the animal models used.

Sbrana *et al.* utilized ground squirrels to test the efficacy of ST-246 against a MPXV challenge [21]. The authors used 100 pfu of MPX-ZAI-1970 (200 × LD_50_) via a subcutaneous route of inoculation. Squirrels (8–9 per group) were divided into five treatment groups; drug was given either at 0 hours of infection, 24 hours, 48 hours, 72 hours or 96 hours p.i. 100 mg/kg of drug was given once a day for 14 days. Two animals in each group were sacrificed at day 7 to measure objective morbidity; the remainder of the animals were used to calculate survival rates. Animals in the placebo group, that were not given ST-246, showed signs of illness beginning on day 4 and all died between days 6–9. Signs of disease included lethargy, anorexia, nosebleeds, and terminal respiratory distress. At day 7, a sampling of placebo-treated animals exhibited significant leukocytosis, transaminitis, and coagulopathy; almost 10^5^ pfu/mL of infectious monkeypox was found in blood; at this time, between 10^7^ and 10^8^ pfu /mL of infectious MPXV was observed in 10% organ homogenates of liver, spleen and lung. Animals treated on days 0, 24, 48 or 72 hours, before symptomatic disease onset, all survived infection and showed no signs of disease. At day 7, in a sampling of animals treated at hour 0, 24, 48 or 72 p.i., no virus was found in the liver, spleen, lung, or blood; although some abnormal values were apparently recorded, no clear trends in leukocytosis, transaminitis or coagulopathy were noted with delay in treatment onset. In animals initiating treatment at 96 hours p.i., concurrent with symptomatic disease onset, 67% of animals survived infection. 2/4 survivors showed signs of disease. In those animals that succumbed to infection, ST-246 prolonged the time to death; the mean time to death was day 7 for animals receiving placebo and day 13 for those receiving ST-246 in the 96 hour p.i. treatment group. The sampling of animals at day 7, initiating ST-246 at 96 hour p.i., demonstrated lower levels of viremia (∼3 log decrease) and ∼5 logs less virus in liver, spleen and lungs than that seen on the placebo treated animals at day 7. Although some evidence of transaminitis was present, leukocytosis and coagulopathy were not observed in this treatment group. Pathologic examination of tissues in general showed greater tissue necrosis in animals treated at later times p.i. This study was able to demonstrate a survival benefit in animals treated prior to, or at the onset of disease symptoms, in a disease model that has a time course attenuated with respect to what is seen in human disease.

Schultz *et al.* infected African dormice with a lethal challenge of Congo Basin clade virus MPXV-ZAI-79 via an IN route of infection to evaluate the efficacy of Cidofovir as post exposure prophylaxis [16]. Four hours post intranasal infection with 75, 4 × 10^3^, or 5 × 10^3^ pfu of MPXV, animals were intraperitoneally administered 100 mg/kg cidofovir (the calculated LD_50_ for the dormouse MPXV model was 12 pfu). Aggregate data from all challenges showed animals treated with cidofovir had a mortality rate of 19% (7/36), whereas vehicle treated animals all (41/41) succumbed to disease. Treatment initiation at later times p.i. was not evaluated; effects on viral load or histopathologic changes were not reported.

As inbred mice have historically shown little disease symptomatology or pathogenesis post monkeypox infection, Stabenow *et al.* utilized a laboratory mouse strain lacking STAT1 (C57BL/6stat-/-), which has been found to be sensitive to a range of viruses including SARS, murine norovirus 1, respiratory viruses, dengue virus and MPXV [19,22–25]. These animals are deficient in their ability to transcribe many of the Type I and Type II receptor interferon response genes. The authors used the Congo Basin clade virus MPX-ZAI-79, evaluated disease and the protective efficacy of CMX001 and ST246. In untreated mice, 0% mortality was observed with 4.7 pfu challenge, 90% mortality with 470 pfu of virus and 100% mortality with 4,700 pfu. Over 25% total body weight loss, and mortality was observed on or prior to day 10 p.i. in untreated animals. Animals in the treatment studies were subsequently challenged with 5,000 pfu via an IN infection. Animals were then treated with 10 mg/kg of CMX001 by gastric gavage on the day of challenge followed by every other day with 2.5 mg/kg until day 14 p.i. All C57BL/6 stat-/- mice that were treated with drug survived infection, demonstrated <10% body weight loss between days 10 and 20, and developed a serologic response to monkeypox. Similarly, mice treated daily, starting at the day of virus challenge, with 100mg/kg of ST246 for 10 days also survived infection and manifest <10% body weight loss between days 10 and 20. In this system, antiviral treated animals rechallenged with monkeypox at day 38 post initial infection (at least 10 days post reinitiation of steady weight gain), manifest 20% mortality. The model—again one with a short disease course—is useful for demonstrating immediate post exposure efficacy of antiviral treatment in the absence of a functioning interferon response system. Additionally, in this animal model system, perhaps due to the immune defect, a monkeypox protective immune response was not elicited in all animals receiving antiviral treatment. This observation merits further observation in other animal model systems.

Smith *et al.* tested the efficacy of ST246 in a prairie dog MPXV model [26]. MPXV challenged prairie dogs have previously been shown to have an asymptomatic period followed by symptoms of disease including lethargy, nasal discharge, inappetence, weight loss and systemic lesion development most commonly between days 9–12. In the current study, animals were inoculated via an IN challenge with the Congo Basin clade virus ROC-2003-358. This is a different strain of MPXV than that used in the previous described studies, but is also a strain belonging to the Congo Basin clade. The challenge dose was 3.8 × 10^5^, equal to 65 × LD_50_ for the prairie dog model. Animals were divided into three treatment groups; prophylactic (day 0), post exposure (day 3) and therapeutic (varying day based on rash onset), and a control vehicle treated group. ST246 was formulated at 30 mg/mL and administered daily, by oral gavage, for 14 days. Animals initiating treatment at day 0 or 3 were protected from death and apparent signs of illness. Animals treated at rash onset had symptoms similar to the placebo control group; however symptoms were less severe in the treated animals. Although all animals treated at rash onset survived infection, animals lost 10–24% of body weight and did develop generalized rash (however, lesions resolved more quickly when compared to untreated prairie dogs in previous studies [13,14]. Although asymptomatic, viable virus was shed sporadically from animals in the prophylaxis and post exposure groups (from two oropharyngeal samples in the day 0 prophylaxis group, and five samples from the day 3 post exposure group). More, sustained virus was detected in the oropharyngeal samplings of the animals in the therapeutic treatment group, but levels were less than the virus levels in the untreated group. 1/4 sham-treated animals survived infection. Signs of disease and viral titers were all increased in this group of animals compared to the animals treated with ST-246. This is the first small animal study where a treatment and survival benefit has been demonstrated when animals are treated at later stages of illness. Initiation of treatment at rash onset is similar to expectations of a human treatment regimen. The observation of virus shedding after treatment cessation in the prophylactically or post exposure treated animals merits further study to assess whether this reflects viral resistance or a blunted and delayed immune recognition and ultimate clearance of virus.

## 4. Discussion

Animal models permit an advance beyond what can be gleaned from tissue culture evaluation of an antiviral effect. The evaluation of an antiviral, in the context of a host with a functioning immune system, enables better understanding of therapeutics’ potential efficacy. The evaluation of an antiviral in the context of an impaired immune system enables better understanding of therapeutic use in a particular immunosuppressed population. Pathogen host range, especially if not a simple issue of receptor utilization, can confound the ability to interpret, and extrapolate to the human, some of the nuances of the host pathogen interaction and prediction of potential human therapeutic benefit. Of the small animal models used to evaluate antiviral efficacy, all have used stringent virus challenges (all greater than 10 × LD_50_) and shown survival benefit. Routes of infection have used methods that attempt to simulate potential human routes of infection and resultant human illness courses. Given the uncertainties of what a human infectious or lethal monkeypox dose is, it is difficult to extrapolate the potential “best fit” of any of these models for human disease. The clinical time course of disease in the prairie dog model, however, has a temporal relationship that is close to what has been described with human systemic orthopoxvirus (variola or monkeypox) disease. However, a limitation of the prairie dog and some of the other described animal systems, with the exception of the mouse model, is a paucity of immune reagents. There are a handful of antiviral compounds which show promise in these small animal models using monkeypox virus as the challenge. Additional studies evaluating treatment benefit when used in later stages of disease, their effect on elicitation of a protective immune response, evaluation of antiviral resistance, and their effect on viral shedding will improve our understanding of how they may be used in treatment of human disease, or in response to epidemic disease.

## Figures and Tables

**Table 1:** Animal models using monkeypox virus (MPXV) as a challenge

	**Genetic Diversity**	**Immuno-reagent Availability**	**MPXV Strain Used**	**Inoculation Route**	**Dosage Used (pfu)**	**Days until Symptoms**	**Rash Development (days)**	**LD_50_**	**Mortality (%)**	**Time to Death**
Guinea pigs [2]	Outbred	Medium	WA	Intracardiac, IN, oral or FP	Not provided	NA	No rash	NA	0	NA
Golden hamsters [2]	Outbred	Medium	WA	Intracardiac, oral, IN, scarified skin	1.5–5.9 × 10^7^	NA	No rash	NA	0	NA
Adult rabbits [2]	Outbred	High	WA	IV	10^7^	5–6	Extensive disseminated rash	__	8	One animal died one month post infection due to cachexia
				Scarified skin/intradermal	10^5^	Not provided	Localized lesion in addition to disseminated rash for some animals	NA	0	NA
				Oral	>10^9^	NA	No rash	NA	0	NA
10 day old rabbits [2]	Outbred	High	WA	Oral	10^6^	4–6	Disseminated rash	__	85	4–14 days
				IN	10^6^	Not provided	No rash	__	83	4–5 days
Adult white rats [2]	Outbred	Medium	WA	IV, IN, cutaneous	10^1^ to 10^3^	NA	No rash	NA	0	NA
1–3 day old white rats [2]	Outbred	Medium	WA	IN	10^1^ to 10^3^	Not provided	No rash	__	100	5–6 days
Cotton rats [6]	Outbred	Medium	WA	IV	10^5^	Not provided	Not provided	__	100	4–5 days
				IN	10^5^	Not provided	Not provided	__	50	4–5 days
Multimammate rats [8]	Outbred	Low	WA	IN, IP	Not provided	Not provided	Not provided	__	Not provided	Not provided
Common squirrel [9] (*Sciurus vulgaris)*	Outbred	Low	CB	IN, oral, scarification	10^6^	1–5	No rash	__	100	7–8 days
African squirrels (six species) [10]	Outbred	Low	CB	IN	10^5^, 10^6^	Not provided	No rash	__	100	Not provided
					10–10^4^	16–25 until rash	Rash on non- fur-bearing areas of skin in a few squirrels	__	Varied based on squirrel species	Not provided
				Oral	10^5^	16–25 until rash	Rash on non- fur-bearing areas of skin in a few squirrels	__	Not provided	Not provided
				Cutaneous	10^5^	Not provided	Localized lesion at inoculation site	__	Not provided	Not provided
Ground squirrel [11,12]	Outbred	Low	WA	IP, IN	10^5.1^	4–5	No rash	__	100	IP: 6–7 days IN: 8–9 days
			WA, CB	Subcutaneous	100	3–5	No rash	0.35 pfu CB 0.46 pfu WA	100	6–11 days
Prairie dogs [13–15]	Outbred	Low	WA	IP	10^5.1^	4	No rash	__	100	8–11 days
				IN	10^5.1^	4	Lesions on lips/tongue	__	60	11–14 days
			WA, CB	IN	10^4.5^	9–12 days until rash	Disseminated rash	WA: 1.29 × 10^5^ pfu CB: 5.9 × 10^3^ pfu	WA: 0 CB: 25	CB: 13 days
				Scarification	10^4.5^	9–12 days until rash	Disseminated rash	__	WA: 0 CB: 50	CB: 11–12 days
8 day old white mice [2]	Not specified	High	WA	FP	6 × 10^2^	Not provided	No rash	__	100	Not provided
				IP	1.2 × 10^6^	Not provided	No rash	__	100	Not provided
				IN	1.2 × 10^6^	Not provided	No rash	__	100	Not provided
				ID	1.2 × 10^6^	Not provided	No rash	__	50	Not provided
				Oral	1.2 × 10^6^	Not provided	No rash	__	40	Not provided
12 day old white mice [2]	Not specified	High	WA	Oral	1.2 × 10^6^	Not provided	No rash	__	14	Not provided
15 day old white mice [2]	Not specified	High	WA	IN	1.2 × 10^6^	Not provided	No rash	__	100	Not provided
BALB/c and C57BL/6 lab mice [17]	Inbred	High	WA, CB	IN, FP	10^5^	6	No rash	NA	0	NA
SCID BALB/c lab mice [18]	Inbred	High	WA, CB	IP	10^5^	5–7	No rash	__	100	9–11 days
C57BL/6stat-/- lab mice [19]	Inbred	High	CB	IN	47	No symptoms except weight loss	No rash	Females: 213 pfu Males: 47 pfu	25–50	12–21 days
					4700	No symptoms except weight loss	No rash	Females: 213 pfu Males: 47 pfu	100	9 days
CAST/EiJ wild-derived lab mice [20]	Inbred	High	CB	IN	10^4^	Not provided	No rash	680 pfu	100	8–10 days
				IP	10^3^	Not provided	No rash	14 pfu	100	6–8 days
			WA	IN	10^4^	Not provided	No rash	7600 pfu	50	9 days
Dormice [16]	Outbred	Low	CB	FP	1.4 × 10^4^	Not provided	No rash	__	92	7–10 days
				IN	2	3	No rash	12 pfu	38	12.3 ± 5 days
				IN	200–2000	3 days	No rash	12 pfu	100	7.9 ± 1 and 8.7 ± 1
